# Eicosapentaenoic and Docosahexaenoic Acid-Enriched High Fat Diet Delays Skeletal Muscle Degradation in Mice

**DOI:** 10.3390/nu8090543

**Published:** 2016-09-03

**Authors:** Nikul K. Soni, Alastair B. Ross, Nathalie Scheers, Otto I Savolainen, Intawat Nookaew, Britt G. Gabrielsson, Ann-Sofie Sandberg

**Affiliations:** 1 Division of Food and Nutrition Science, Department of Biology and Biological Engineering, Chalmers University of Technology, Gothenburg SE-41296, Sweden; alastair.ross@chalmers.se (A.B.R.); nathalie.scheers@chalmers.se (N.S.); otto.savolainen@chalmers.se (O.I.S.); bggabr@gmail.com (B.G.G.); ann-sofie.sandberg@chalmers.se (A.-S.S.); 2 Division of Systems and Synthetic Biology, Department of Biology and Biological Engineering, Chalmers University of Technology, Gothenburg SE-412 96, Sweden; intawat@chalmers.se; 3 Department of Biomedical Informatics, College of Medicine, University of Arkansas for Medical Sciences, Little Rock, AR 72204, USA

**Keywords:** eicosapentaenoic acid (EPA)/Docosahexaenoic acid (DHA), obesity, skeletal-muscle metabolism, mitochondrial β-oxidation, transcriptome

## Abstract

Low-grade chronic inflammatory conditions such as ageing, obesity and related metabolic disorders are associated with deterioration of skeletal muscle (SkM). Human studies have shown that marine fatty acids influence SkM function, though the underlying mechanisms of action are unknown. As a model of diet-induced obesity, we fed C57BL/6J mice either a high fat diet (HFD) with purified marine fatty acids eicosapentaenoic acid (EPA) and docosahexaenoic acid (DHA) (HFD-ED), a HFD with corn oil, or normal mouse chow for 8 weeks; and used transcriptomics to identify the molecular effects of EPA and DHA on SkM. Consumption of ED-enriched HFD modulated SkM metabolism through increased gene expression of mitochondrial β-oxidation and slow-fiber type genes compared with HFD-corn oil fed mice. Furthermore, HFD-ED intake increased nuclear localization of nuclear factor of activated T-cells (Nfatc4) protein, which controls fiber-type composition. This data suggests a role for EPA and DHA in mitigating some of the molecular responses due to a HFD in SkM. Overall, the results suggest that increased consumption of the marine fatty acids EPA and DHA may aid in the prevention of molecular processes that lead to muscle deterioration commonly associated with obesity-induced low-grade inflammation.

## 1. Introduction

The World Health Organization (WHO) estimates that chronic inflammatory conditions accounts for more than 17 million deaths every year [1]. Obesity is considered one of the main underlying factors for chronic inflammation and has almost doubled since 1980 and now over 1.4 billion adults worldwide are obese. Obesity, once considered a problem only in developed countries, is now also a major problem in low- and middle-income countries. Parallel to the increase in obesity, diseases associated with chronic inflammation such as cardiovascular diseases and type 2 diabetes are also rising [1]. One of the major concerns associated with low-grade inflammation is sarcopenia—loss of muscle mass. Generally, after the age of 50 the muscle mass reduces at a rate of up to 1%–2% annually, while fat mass increases [2,3]. At the same time, muscle strength drops at the rate of 1.5% annually between the age of 50 and 60 years and at the rate of 3% thereafter [4]. Obesity accelerates age-related muscle deterioration [5] exacerbating the risk for type 2 diabetes (T2D) by approximately 30% [6]. The negative feedback loop defined as the combination of excess weight-gain and reduced muscle mass, strength and performance is known as sarcopenic obesity [7]. Further suggestions of a role for inflammation in sarcopenia includes an association between increased TNF-α [8] and Il-6 [9] and decreased muscle mass and strength. 

Skeletal muscle (SkM) constitutes about 40% of total body mass in adult lean men and is an adaptable tissue in response to changes in lifestyle such as diet and physical training [10,11]. SkM is one of the major sites of glucose metabolism, accounting for about 30% of postprandial glucose disposal [12]. This makes SkM a crucial organ for maintaining healthy glucose concentrations in the body, with loss of muscle mass also associated with increased type 2 diabetes risk. Changes to diet may impact on SkM and use of fish oil rich in the fatty acids eicosapentaenoic acid (EPA) and docosahexaenoic acid (DHA) have been proposed to improve SkM metabolism [13]. An intervention with marine fatty acids improved muscle mass and function in older adults [13] and animal studies suggest a role for marine fatty acids in SkM anabolism and increased protein synthesis [14,15]. In addition, marine fatty acids have anti-inflammatory activity in animals and humans [16], which may reduce the loss of inflammation-mediated SkM mass in older adults. Supplementation with EPA and DHA, the two main marine fatty acids, could be the basis for a simple, safe and low-cost solution for preventing and mitigating negative changes to SkM metabolism. For such a strategy to be credible, it is crucial to understand the underlying signaling mechanisms mediated by EPA and DHA on SkM.

Our previous intervention studies showed that mice fed high fat diets (HFDs) supplemented with herring improve muscle mass compared with mice fed HFD supplemented with beef [17,18]. In several randomized control trials in humans, marine fatty acids improve muscle volume, a surrogate marker for improved muscle performance [13,19,20]. In order to build on these results and determine if EPA and DHA, or other components in fatty fish, are responsible for improving muscle metabolism and to determine what the underlying mechanisms could be, we conducted a follow-up trial on earlier work with fatty fish [17,18]. Here we have used transcriptomics to explore how replacing a commonly used source of dietary fat, corn oil with purified EPA and DHA interacts with gastrocnemicus skeletal muscle (gSkM) gene expression, in order to investigate how EPA and DHA can affect muscle deterioration.

## 2. Materials and Methods

### 2.1. Animal Experiment

Six-week old male C57BL/6 J mice (Harlan BV, The Netherlands) were grouped 5–6 mice/cage, and were housed in a temperature and humidity-controlled environment with 12 h light/dark cycle for three weeks to acclimatize to the facility. At nine weeks of age, mice were switched from a normal mouse chow to one of three intervention diets: high fat diet with EPA and DHA (HFD-ED), a high fat diet with corn oil (HFD-corn oil) or the same mouse chow as during the acclimatization period (control group). Mice were maintained on these diets for 8-weeks (see Diets for details). After 8-weeks the animals were anesthetized by intraperitoneal injection of sodium pentobarbital (>60 mg/kg) and blood was collected from the heart followed by cervical dislocation. Plasma was obtained by centrifugation at 4 °C of EDTA whole blood (10 min; 10,000× *g*). The tissues were weighed and snap-frozen in liquid nitrogen immediately after dissection. Both, tissues and plasma were stored at −80 °C until further analysis. The animal experiments were approved by the Animal Ethics Committee at the University of Gothenburg (Dnr: 253-2009) [21]. 

### 2.2. Diets

The mice were fed a standard control chow during the 3-weeks acclimatization period. The mice did not differ in body weight at the start of the study (Table 1) and the body weights were measured weekly during the study. We removed two mice from the control group due to unsocial behavior, thus there were *n* = 9 mice in control and *n* = 12 mice in either HFD group. HFD were used to induce obesity in the mice. The two HFD used in the study differed only in their fatty acid composition. The reference HFD was prepared with 5 weight/weight (*w*/*w*)% corn oil, while the treatment HFD was prepared with 2 *w*/*w*% purified EPA and DHA (EPAX AS Lysaker, Norway) plus 3 *w*/*w*% corn oil. The control diet provided 24 energy% (E%) as protein, 12 E% as fat and 65 E% as carbohydrates, whereas the two HFD contained 25 E% as protein, 32 E% as fat and 44 E% as carbohydrates (Table 2). The diets were prepared by Lantmännen AB (Kimstad, Sweden). Access to water and diet was ad libitum and the diets were changed three times per week throughout the study.

### 2.3. Muscle Composition

Gastrocnemicus skeletal muscles were dissected and weighed directly after sacrifice. Plasma total triglyceride and cholesterol was measured enzymatically with Konelab Autoanalyser version 2.0 at the Department of Clinical Chemistry, Sahlgrenska University Hospital, Gothenburg, Sweden. Clinical chemistry procedures have been previously described in detail [21].

### 2.4. RNA Isolation and Quality Assurance

Four mice, representative from each diet group on the basis of body weight, plasma triglyceride and plasma cholesterol levels were selected for RNA isolation and microarray analysis (Supplemental Table S1). Total RNA from gSkM was purified using the RNeasy^®^ Plus Universal Mini kit (Qiagen Nordic, Sollentuna, Sweden). RNA samples were quantified spectrophotometrically (NanoDrop 2000c UV-Vis Spectrophotometer, Thermo Scientific, Wilmington, NC, USA) and the quality of the RNA was controlled by the ratio of 28S and 18S rRNA (RNA 6000 Nano LabChip for Agilent 2100 Bioanalyzer (Agilent Technologies, Santa Clara, CA, USA).

### 2.5. Western Blot Analysis

Nuclear and cytosolic extracts were prepared using NE-PER Nuclear and Cytoplasmic Extraction Reagents kit (78833, Thermo Fisher Scientific). Western blot analysis was performed as described earlier [22]. Briefly, the protein concentration was measured in duplicate using the Pierce BCA Protein Assay kit (Thermo Fisher Scientific) for equal protein loading to the SDS-PAGE. After transfer of the proteins to the membranes, the membranes were stained with 0.5% Ponceau S (Merck Chemicals, Darmstadt, Germany) to visualize the protein transfer. For the detection of specific proteins, the following primary antibodies were used: Rb-anti-m acetyl-CoA carboxylase (Acc; 3662; Cell Signaling Technology), Rb-anti-m phospho-acetyl-CoA carboxylase (P-Acc; Ser79, 3661; Cell Signaling Technology), Rb-anti-m troponin c1 (Tnnc1; sc-20642, Santa Cruz Biotechnology), Rb-anti-m Nuclear factor of activated T-cells (Nfatc4; sc-13036, Santa Cruz Biotechnology). The secondary antibodies were: HRP-conjugated Horse-anti-m IgG (7076; Cell Signaling Technology), Goat-anti-m IgG (sc-2020; Santa Cruz Biotechnology) and Rb-anti-m IgG (7074; Cell Signaling Technology). As loading control for each western blot, either glyceraldehyde 3-phosphate dehydrogenase (anti-Gapdh; sc-47724; Santa Cruz Biotechnology), or lamin (anti-lamin A/C, sc-6215; Santa Cruz Biotechnology) was used. Western blots for the protein Acc, P-Acc and Tnnc1 are in supplemental Figure S1.

### 2.6. Microarray—Data Acquisition and Analysis

RNA was labeled and hybridized to MouseWG-6_V2.0 Expression BeadChip (R3_11278593_A; Illumina, CA, USA) that contains 45,281 transcripts, at the SCIBLU Genomics core facility (Swegene Centre for Integrative Biology at Lund University, Sweden). The content of this BeadChip is derived from the National Center for Biotechnology Information Reference Sequence (NCBI RefSeq) database (Build 36, Release 22) [23], supplemented with probes from the Mouse Exonic Evidence Based Oligonucleotide (MEEBO) [24] set, as well as example protein-coding sequences from RIKEN FANTOM2 database [25]. The raw and normalized data is available in SOFT format at the Gene Expression Omnibus database under Accession Number GSE76361.

The Illumina bead array files were imported to GenomeStudio Gene Expression (GSGX) software 1.9.0. The un-normalized expression data (fluorescence intensities) were extracted for the mean intensity of arrays. R Studio software (Version 3.2.3, © 2015 RStudio, Inc., Boston, MA, USA) was used for microarray data analysis. The data was quantile normalized and variance-stabilizing transformation (VST) was performed using the default setting of *lumiExpresso* function from the lumi package [26]. The top 100 differentially expressed genes regulated by HFD-ED compared with HFD-corn oil are provided in supplemental Table S2. The Empirical Bayes method from the limma package was then applied to the signals to calculate moderated *t*- and *F*-statistics, log odds and differential expression for comparisons between the diets from skeletal muscle tissue [27]. The Generally Applicable Gene-Set/Pathway Analysis (GAGE) Bioconductor package was used for gene-set enrichment analysis (GSEA) for functional inference [28]. Before implementing the gage function, canonical signaling and metabolic pathways from the KEGG mouse database was prepared for better-defined results. For the microarray data, false discovery rate (FDR) adjusted *p*-values were calculated and FDR adjusted *p*-value < 0.05 was considered significant. The Pathview package was used to visualize the data, where the *pathview* function downloads KEGG graph data and renders a pathway map, based on experimental results [29].

### 2.7. Fatty Acid Analysis

Approximately 100 mg gSkM tissue was weighed, freeze-dried and extracted using the Folch total lipid extraction method [30]. Briefly, 5 mL chloroform–methanol (2:1 *v/v*) was added to the samples followed by sonication at 40 Hz for 30 min at RT (Branson 8510, Branson Ultrasonics Corp, CT, USA). One mL of physiological saline (0.9% NaCl) was mixed with the samples followed by brief vortexing and centrifugation for 5 min at 3000 rpm (1700× *g*), to separate the two phases. The organic lower phase was collected and the remaining aqueous phase was re-extracted with 1.5 mL chloroform. The two extracted phases were combined and evaporated under N_2_ and re-dissolved in 1 mL isopropanol. All solvents were from Sigma-Aldrich, Sweden. 

Solid phase extraction (SPE) method was used to separate lipid classes [31]. Internal phospholipid (C17:0) and triglyceride (C19:0) standards (Nu-Chek prep, Inc., Elysian, MN, USA) were added to the samples. Hexane conditioned aminopropyl SPE columns were loaded with 1 mL of extracted SkM lipid samples. Neutral lipids, free fatty acid and phospholipids were eluted with chloroform–isopropanol (2:1), diethylether-2% acetone and methanol, respectively and evaporated under N_2_. Dried SPE fractions were methylated by overnight incubation with 1 mL 10% acetyl chloride in methanol and 1 mL toluene. After methylation, 0.3 mL water and 4 mL petroleum ether was added to the samples and the resulting two phases were separated by 3 min centrifugation at 3000× *g*. The upper phase was then removed, evaporated under N_2_ and re-dissolved in 250 μL isooctane [32].

The fatty acid methyl esters (FAMEs) were analyzed by gas chromatography mass spectrometry (Focus GC, ISQ, Thermo Fischer Scientific, USA) using a ZB-WAX column (30 × 0.25 mm I.D., 0.25 μm) (Phenomenex, UK). One µl of sample was injected in splitless mode. The injector temperature was 240 and helium was used as the carrier gas with the flow of 1 mL/min. Oven temperature was as follows: 50 °C 1.5 min, ramped to 180 °C at 25 °C/min where held for 1 min, ramped to 220 °C at 10 °C/min, held for 1 min and ramped to 250 °C at 15 °C/min and held for 3 min. The transfer line was kept at 250 °C and ion source at 200 °C. Electron impact ionization (70 eV) was used and the FAMEs were detected by scanning a mass range from 50 to 650 *m/z*. An external FAME standard mix GLC 463 (Nu-Chek prep, Inc., Elysian, MN, USA) was used for identification of peaks in gSkM samples. Fatty acids were quantified against internal standards, summed and expressed as mg/g dry gSkM biomass and as a percentage of total lipid fractions.

### 2.8. Statistical Analysis of Western Blot and Fatty Acid Analyses

Differences in SkM western blot and fatty acid analyses were tested using ANOVA with Tukey’s honest significant difference (Tukey-HSD) post-hoc test. Statistical significance between the protein groups was calculated with an unpaired 2-tailed Student’s *t*-test. A *p*-value < 0.05 was considered statistically significant. Statistical calculations were performed using Microsoft^®^ Excel^®^ for Mac 2011 version 14.5.8 and R software (Version 3.2.3) under RStudio interface. All data are presented as mean ± SEM.

## 3. Results

### 3.1. Effect of EPA and DHA vs Corn Oil on gSkM Weight and Transcriptome

Delayed weight gain was observed in HFD-ED compared with HFD-corn oil fed animals (Table 1). Moreover, relative adiposity was lower in HFD-ED compared with HFD-corn oil fed mice [21]. Principle component analysis of the gSkM transcriptome did not detect any outliers in the data (Supplemental Figure S2a) indicating good analytical reproducibility. To visualize the global effects of different diets on gSkM, differential expression of the genes was performed on the following comparisons: HFD-ED versus control diet, HFD-corn oil versus control diet and HFD-ED versus HFD-corn oil. Venn diagrams show the number of differentially expressed genes (DEGs) corrected for multiple testing by Benjamini and Hochberg at *p*-value < 0.001 (supplemental Figure S2b). The total number of DEGs was 365, 129 and 383 for the comparison HFD-ED versus control diet, HFD-corn oil versus control diet and HFD-ED versus HFD-corn oil, respectively. Differential gene expression analysis found that the different fat composition of the two HFDs affected gene expression in gSkM differently when compared to the control group (Supplemental Figure S3).

### 3.2. gSkM Fatty Acid Composition

Neutral lipids: The total amount of neutral lipids was significantly higher in the mice fed HFD-corn oil compared to mice fed the other two study diets (Table 3). The concentration of fatty acid C12:0 was higher in HFD-corn oil fed animals compared with both HFD-ED and control diets (Table 3). C14:0 was higher in HFD-corn oil fed animals compared with control diet but there was no difference compared with HFD-ED fed animals. Fatty acids C16:0, C18:1 and C18:2 *n*-6 (linoleic acid) were higher in HFD-corn oil fed animals compared with mice fed HFD-ED or control diets, whereas no difference was observed between the control and HFD-ED fed animals. C22:6 *n*-3 (DHA) was higher in the HFD-ED fed animals compared to the control diet.

Free fatty acids: Free fatty acids (FFA) mainly comprised of saturated and monounsaturated fatty acids including C16:0, C19:0, C16:1 and C18:1 (Table 3). Apart from C12:0, which was higher in HFD-ED fed animals, the remaining FFAs were higher in HFD-corn oil fed animals compared with the other two diets. The concentration of C20:3 *n*-3 was higher in the HFD-corn oil fed animals compared with HFD-ED and the concentration did not differ compared with control diet. As expected, C22:6 *n*-3 concentrations were higher in the HFD-ED fed animals compared with HFD-corn oil. The total amount of FFAs in gSkM did not differ between the different groups. 

Phospholipids: The concentration of C14:0 was significantly higher in the HFD-ED fed animals compared with the other two diets (Table 3). The amount of C18:1 was significantly lower in the HFD-ED fed animals compared with HFD-corn oil fed animals. The amount of C20:4 *n*-6 (arachidonic acid) was lower in HFD-ED fed animals compared with either HFD-corn oil or control diet fed animals. The amount of *n*-3 PUFA C20:5 *n*-3 (EPA) did not differ for any of the diets, with only very low concentrations measured, whereas the concentrations of C22:5 *n*-3 (DPA) and C22:6 *n*-3 (DHA) were significantly higher in the animals fed HFD-ED compared with animals fed HFD-corn oil but the expected increase in EPA was not found. The total phospholipid content was significantly lower in the HFD-ED fed animals compared with control diet fed animals but did not differ from that found in gSkM from the animals fed HFD-corn oil. 

### 3.3. Increased Mitochondrial B—Oxidation and Oxidative Phosphorylation in the Gskm of Mice Fed HFD-ED

Energy metabolism is a biochemical process to generate energy from nutrients via aerobic or anaerobic pathways. KEGG pathway analysis suggested that HFD-ED intake led to an increased regulation of energy metabolism relating to lipid metabolism, Krebs cycle and oxidative phosphorylation, leading to increased capacity for ATP production in gSkM compared with HFD-corn oil fed animals.

**Figure 1:**
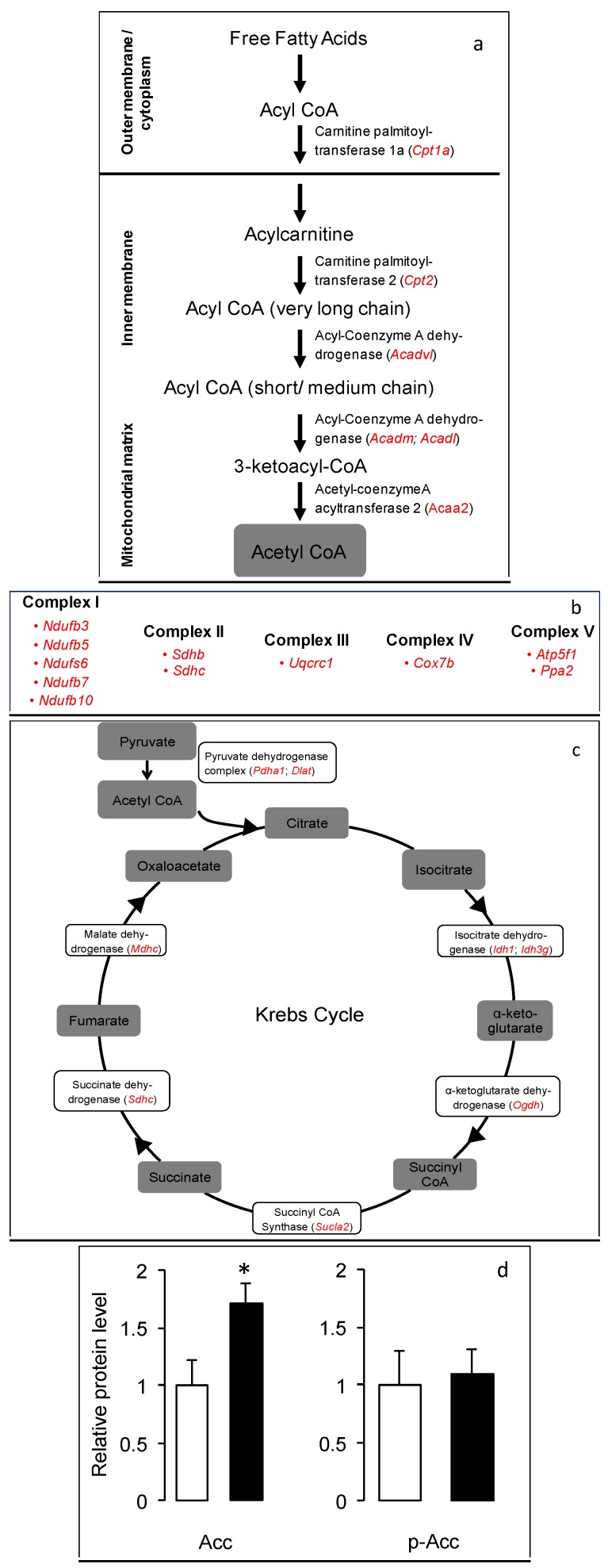
Pathway analysis of the gSkM transcriptome for the comparison of HFD-ED and HFD-corn oil fed mice. The illustration is based on the Kegg pathway database (http://www.genome.jp/kegg/pathway.html). (**a**) Fatty acid β-oxidation: Genes highlighted in red are upregulated in gSkM of mice fed HFD-ED compared with HFD-corn oil; (**b**) Electron transport chain: Genes highlighted in red are upregulated in gSkM of mice fed HFD-ED compared with HFD-corn oil; (**c**) Krebs Cycle: Genes highlighted in red are upregulated in gSkM of mice fed HFD-ED compared with HFD-corn oil; (**d**) Relative Acc protein levels in HFD-ED (white bars) fed mice was lower than HFD-corn oil (black bars) and there was no difference in the phosphorylation of Acc (p-Acc) protein after 8-weeks diet intervention. * Different at *p* < 0.05.

Eight gene transcripts were upregulated by HFD-ED compared with the HFD-corn oil fed mice (Figure 1a). Functionally, the enzymes in the β-oxidation pathway facilitate the breakdown of fatty acids to form acetyl-CoA, which enters the Krebs cycle. The upregulated gene products, which are responsible for transporting fatty acids into the mitochondria, were two carnitine palmitoyltransferases (*Cpt1a* and *Cpt2*). In addition, RNA transcription of genes responsible for the subsequent degradation of fatty acids within the mitochondrial matrix producing acetyl-CoA including short/medium chain acyl-CoA dehydrogenase (*Acadm*), long chain acyl-CoA dehydrogenase (*Acadl*), very long chain acyl-CoA dehydrogenase (*Acadvl*), acetyl-CoA acyl transferase 2 (*Acaa2*), enoyl-CoA delta isomerase 1 (*Eci1*) and aldehyde dehydrogenese (*Aldh2*), were upregulated by the HFD-ED diet.

Pathway analyses showed a significant increase in the oxidative phosphorylation pathway in gSkM from the HFD-ED fed mice compared with HFD-corn oil animals (Figure 1b). Several genes involved in electron transport complex I-V were upregulated by HFD-ED fed mice compared with HFD-corn oil. Genes from the NADH dehydrogenase family that constitutes complex I (*Ndufa11*, *Ndufb3*, *Ndufb5*, *Ndufb7*, *Ndufb10* and *Ndufs6*); two succinate dehydrogenases (*Sdhb* and *Sdhc*) from complex II; ubiquinol-cytochrome c reductase core protein 1, cytochrome c oxidase subunit VIIb-Muscle and ATP synthase H+ transporting mitochondrial F0 complex subunit B1 (*Uqcrc1*, *Cox7b* and *Atp5f1*) constituting complex-III, IV and complex-V respectively, were upregulated.

HFD-ED upregulated the Krebs cycle in mice gSkM compared with HFD-corn oil (Figure 1c). The mitochondrial event consumes Acetyl-CoA producing NADH, FADH_2_, GTP and the by-product CO_2_. NADH and FADH_2_ are in turn used by oxidative phosphorylation to generate ATP. Genes for key proteins in this metabolic pathway that were upregulated by EPA and DHA include pyruvate dehydrogenase E1 alpha 1 (*Pdha1*) and dihydrolipoamide S-acetyl transferase (*Dlat*), which converts pyruvate to Acetyl-CoA. HFD-ED upregulated several Krebs cycle genes including two kinds of isocitrate dehydrogenases (*Idh1* and *Idh3g*), oxoglutarate dehydrogenase (*Ogdh*), Succinate-CoA ligase GDP forming alpha subunit 2 (*Sucla2*), succinate dehydrogenase subunit a (*Sdha*) and malate dehydrogenase 2 (*Mdh2*), when compared with HFD-corn oil. There was no difference in the gene expression of *Acacb* (Acetyl-CoA carboxylase beta) but the total protein content of total acetyl-CoA carboxylase (Acc) was markedly lower in the gSkM of HFD-ED fed mice (Figure 1d). However, the phosphorylation levels of Acc did not differ in between the two HFDs (Figure 1d). This suggests increased internalization of fatty acids in mitochondria for potential β-oxidation in gSkM of HFD-ED fed mice rather than synthesis of fatty acids, as seems to be ongoing in HFD-corn oil animals.

### 3.4. Increased Expression of Muscle Contraction Pathway Genes in the Gskm of Mice Fed HFD-ED

**Figure 2:**
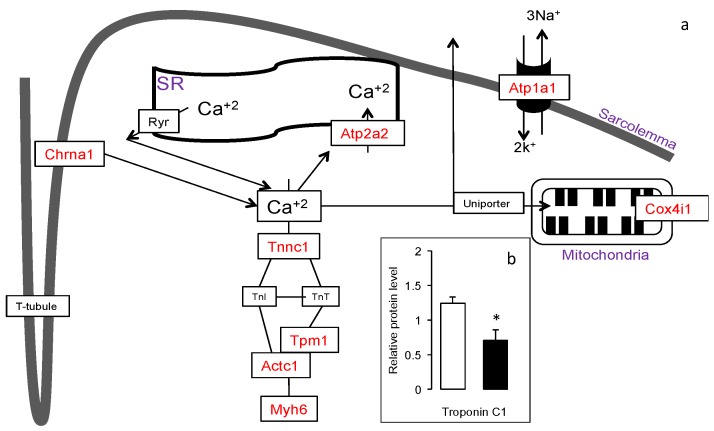
Pathway analysis of the gSkM transcriptome for the comparison HFD-ED versus HFD-corn oil fed mice. The illustration is based on the Kegg pathway database (http://www.genome.jp/kegg/pathway.html). (**a**) Nerve-Muscle contraction: Genes highlighted in red are upregulated in gSkM of mice fed HFD-ED compared with HFD-corn oil; (**b**) Relative Troponin C1 protein level in HFD-corn oil (black bars) fed mice was lower than HFD-ED (white bars). * Different at *p* < 0.05.

Skeletal muscle fibers require stimulation from the neuromuscular junctions acting on the cholinergic nicotine receptor on the muscle cells to initiate contraction. These efferent cholinergic nerves are crucial for voluntary control of skeletal muscles. Gene expression of the cholinergic receptor nicotinic α polypeptide 1 (*Chrna1*), insulin growth factor receptor (*Igfr*) and two l-type voltage dependent Ca-channel genes (*Cacna1s* and *Cacnb2)*, two calcium ATPases (*Atp1a1* and *Atp2a2*, a.k.a *Serca* 1 and 2, respectively), were upregulated by HFD-ED compared with HFD-corn oil fed mice (Figure 2a).

### 3.5. Increased Slow-Fiber-Specific Gene Expression Program in the Gskm of Mice Fed HFD-ED

**Figure 3:**
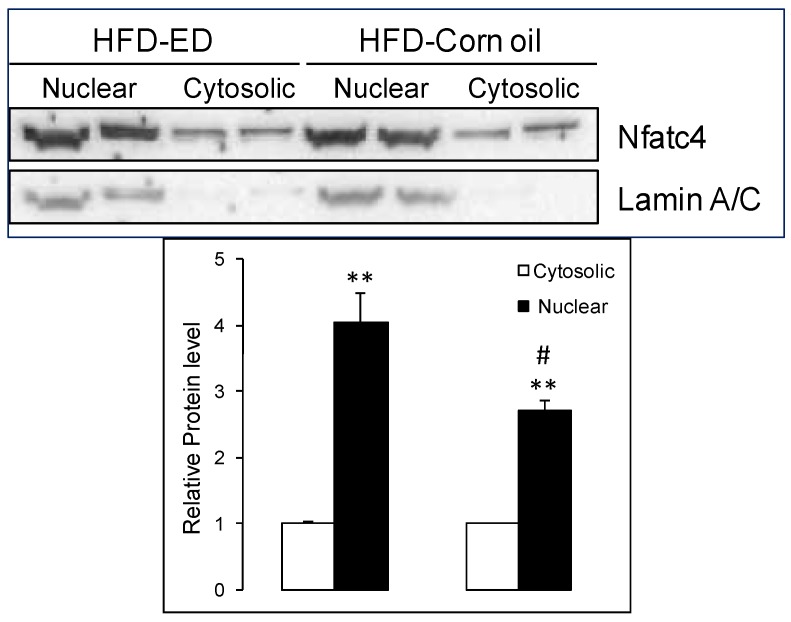
Representative Western blot is shown with LaminA/C loading control. Nuclear (black bars) and cytosolic (white bars) protein extract from the gSkM of HFD-ED (left-panel) and HFD-corn oil (right-panel) were analyzed by Western blot using antibodies for total anti-Nfatc4 antibody and the cytosolic protein levels in the HFD-ED was kept to 1. Different at ** *p* < 0.05 and ^#^ *p* < 0.01.

The calcium mediated calcineurin-Nfat signaling cascade has been suggested to upregulate slow-fiber type gene expression. Differential gene expression of gSkM from the mice fed HFD-ED compared with HFD-corn oil suggested upregulation of several Nfat (*Nfatc1*; *Nfatc2*; *Nfatc4*) isoforms. The slow-fiber type genes troponin C1, cardiac/slow skeletal (*Tnnc1*), tropomyosin 1 alpha (*Tpm1*) and actin alpha 1 (*Actc1*) complex genes and crossbridge myosin heavy polypeptide isoforms (*Myh1*, *Myh2*, *Myh3*, *Myh6*, *Myh7*, *Myh8*) were upregulated in gSkM of HFD-ED fed mice compared with HFD-corn oil animals. Protein levels of *Tnnc1,* a biomarker for slow muscle fiber type, were higher in gSkM of HFD-ED fed mice compared with HFD-corn oil animals (Figure 2b). Furthermore, the gene product forming complex with *Nfatc2* and *Nfact4*, namely myogenin (*Myog*) and GATA Binding Protein 5 (*Gata5*) regulating the transcription of slow-fiber type gene expression was upregulated in the HFD-ED gSkM compared with HFD-corn oil. Nuclear localization of Nfat protein is essential for regulation of the slow-fiber-type gene expression. In the gSkM of HFD-ED fed mice, nuclear protein Nfat levels were significantly higher than HFD-corn oil fed animals (Figure 3). The master regulator of the calcineurin-Nfat signaling cascade, the regulator of calcineurin-2, *Rcan2*, was upregulated and its inhibitor glycogen synthase kinase 3β (*Gsk3*β) was downregulated in mice fed HFD-ED, compared with HFD-corn oil animals. Another transcriptional activator, suggested to be a direct target of marine fatty acids, peroxisome proliferator-activated receptor-γ coactivator 1α (*Ppargc1*α) was upregulated in gSkM of HFD-ED compared with HFD-corn oil fed mice.

## 4. Discussion

**Figure 4:**
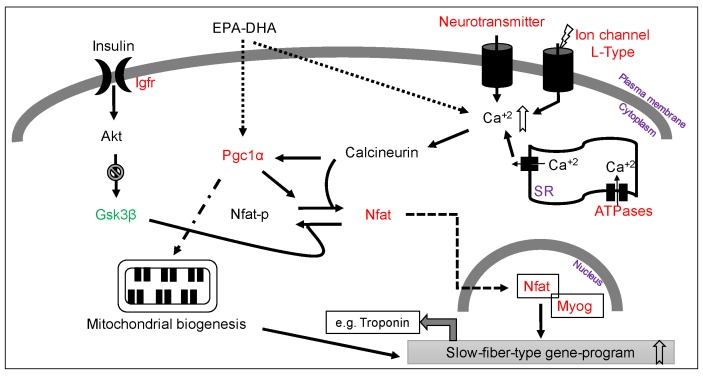
Schematic representation of the role of ED—enriched HFD on the gSkM transcriptome and modulation of ageing and/or obesity-induced sarcopenia in C57Bl/6J mice. Briefly, ED enriched HFD downregulates glucose synthase kinase 3 beta (*Gsk3*β) that phosphorylates nuclear factor of activated T cells (*Nfatc4*) and excludes it from entering into the nucleus. It also increases cytosolic calcium concentrations that in turn regulate calcineurin that then dephosphorylates Nfat allowing its localization in the nucleus. Upon localization, *Nfat* forms complex with myogenin (*Myog*) to turn on slow-fiber-type specific gene-program for e.g., Troponin C1. *Pgc1*α, a known master regulator of mitochondrial biogenesis was also seen upregulated by ED enriched HFD that could act upon multiple targets, one of which regulating dephosphorylation of *Nfatc4* isoforms and its further nuclear localization.

In this study, we have shown that HFD-ED improves muscle metabolism and promotes a switch to slow-fiber type compared to HFD-corn oil. HFD-ED improves metabolism of fatty acids in gSkM by upregulating expression of genes coding for proteins involved in fatty acid β-oxidation, the Krebs cycle and oxidative phosphorylation. Lipid analysis shows that the HFD-ED fed animals had lower total fatty acid content compared to HFD-corn oil and control diet fed animals. Reduced ectopic fat accumulation in HFD-ED compared with HFD-corn oil fed animals suggests a physiological effect of EPA and DHA on muscle metabolic health. Higher expression of the genes involved in muscle contraction was found in gSkM of HFD-ED fed animals compared with HFD-corn oil fed animals. Western blot analysis showed reduced Acc protein levels in gSkM of HFD-ED fed animals compared with HFD-corn oil, confirming gene expression results. Acc is an inhibitor of fatty acid β-oxidation in mitochondria and the lower levels shows increase in mitochondrial fatty acid β-oxidation. In addition, increased Tnnc1 levels suggest increased muscle contraction in gSkM of HFD-ED fed animals compared with HFD-corn oil. Moreover, increased nuclear protein levels of the Nfatc4 isoform strongly suggests up-regulation of slow-fiber-type gene expression in gSkM of HFD-ED fed animals compared with HFD-corn oil fed animals (Figure 4).

The gSkM neutral lipid and free fatty acid profiles reflect the higher amount of DHA in the feed of HFD-ED fed mice, though EPA was not detected in either fraction, in line with our previous findings in liver; that EPA was present in very low amounts, not in proportion to EPA in the diet [21]. There was no substantial proportional difference in gSkM fatty acid composition between the two HFD groups, even though HFD-corn oil fed mice had nearly twice the amount of total neutral lipids. This suggests that ED does not specifically upregulate any of the reactions related to β-oxidation and rather, leads to stimulation of β-oxidation in general, resulting in reduced total neutral lipids in SkM. Although there were several differences in the amount and proportion of free fatty acids and phospholipids, these differences were not quantitatively important aside from the increase DPA and DHA in mice fed HFD-ED but lower EPA in phospholipids fractions [33]. Notably C18:1 was substantially higher in HFD-corn oil fed mice compared with HFD-ED fed mice and this result was reversed for DHA. This could have implications for membrane fluidity within SkM and this may play a role in the observed transcriptome changes.

Previously we found that HFD-ED induced β-oxidation reduced liver fat compared to HFD-corn oil [21], similarly, reduced SkM fat can be due to the increase in β-oxidation and the lower amount of neutral lipids in HFD-ED fed mice. Reduced SkM mitochondrial fatty acid β-oxidation has been proposed as a key feature leading to insulin resistance [34,35] and high fat diets led to downregulation of the group of genes involved in oxidative phosphorylation in human SkM biopsies [36,37,38]. In the gSkM of the HFD-ED fed mice, the expression of rate limiting enzymes in fatty acid β-oxidation in mitochondria, *Cpt1* and *Cpt2* were upregulated compared with HFD-corn oil fed mice. Notably, the protein level of Acc was significantly reduced in gSkM of HFD-ED fed mice compared with HFD-corn oil. Acc regulates fatty acid metabolism by synthesizing malonyl-CoA (building blocks for new fatty acids) but suppresses mitochondrial β-oxidation by inhibiting fatty acid transporter *Cpt1*. Therefore, lower protein levels of Acc suggests entry of fatty acid into mitochondria via *Cpt1* transporter for mitochondrial β-oxidation in gSkM of HFD-ED fed mice compared with HFD-corn oil. A SkM specific isoform of *Acc* (*Acc2*) knockout mice were protected against fat-induced peripheral insulin resistance [39] whereas overexpression of *Cpt1* is sufficient to reduce insulin resistance [40]. Impairments in mitochondrial oxidative capacity in skeletal muscle have been seen in relation to a decrease in energy expenditure [35,41,42]. Thus, increased mitochondrial oxidative capacity in the gSkM of HFD-ED fed animals, supported by increased levels by *Cpt1* and decreased total protein levels of Acc may contribute to increased energy expenditure. Further work on a possible stimulation of energy expenditure by EPA and DHA, possibly via diet-induced thermogenesis is required. 

Ageing and obesity may contribute to the decrease in the contractibility of skeletal muscle. Muscle contraction is regulated by intracellular calcium concentration via the thin filament regulatory proteins troponin and tropomyosin. In the absence of calcium, actin-myosin interaction and subsequently muscle contraction is inhibited. In the gSkM of HFD-ED, two ATPases, namely Na^+^/K^+^-ATPase transporting α2 polypeptide (*Atp1a2*; sodium pump) and ATPase Ca^+2^ transporting skeletal muscle slow switch 2 (*Atp2a2*; *Serca2a*) were upregulated compared with HFD-corn oil. A tissue specific knockout study shows that the sodium pump (*Atp1a2*) is required to prevent fatigue, and systematic analysis of Serca isoforms in calcium transport showed their importance for restoring muscle contractibility [43,44]. Protein levels of the oxidative fiber biomarker Troponin C1 were higher in gSkM of HFD-ED fed mice compared with HFD-corn oil, indicative of increased muscle contraction via control of intracellular calcium in mice fed HFD-ED. Together, these results suggest that HFD-ED contributes to increased calcium release into the sarcoplasm, compared with HFD-corn oil fed mice. 

Calcineurin has been proposed to play a major role in the upregulation of slow-fiber-specific gene expression [45,46,47]. Earlier, it was found that inhibition of calcineurin by cyclosporin-A could result in an increased number of fast fibers in rat skeletal muscle [45]. Activated calcineurin phosphatase capacity is required for dephosphorylation of Nfat proteins, which then localize to the nucleus to initiate slow-fiber-specific gene expression [45,47]. Our data shows that the gSkM of HFD-ED fed animals has markedly higher nuclear levels of Nfat (Nfatc4 isoform) compared with HFD-corn oil, which could contribute to upregulation of the slow-fiber-specific gene expression upon nuclear localization. An earlier study showed abundant myogenin mRNA in slow oxidative muscles and this relationship followed phenotype transition caused by cross-innervation [48]. In HFD-ED fed mice, myogenin (*P_adj_*-value = 0.06) tends to be higher compared with HFD-corn oil fed animals suggesting upregulation of genes for the slower muscle fiber phenotype. We also found that EPA and DHA increased gene expression of *Ppargc1*α, a master regulator of mitochondrial biogenesis leading to red type I fiber, also suggests genetic programing towards stimulating slower muscle fiber [49,50,51,52]. It is known that the products of peroxisomal β-oxidation are also targeted towards mitochondria for oxidation in skeletal muscle and therefore assist in complete lipid disposal [53]. Further support for an effect on muscle fiber type comes from stimulation of insulin growth factor 1 (*Igf*-1), which regulates the *Akt* pathway. Activation of *Akt* phosphorylates *Gsk3*β thereby inactivating it. *Gsk3*β phosphorylates *Nfat* and excluding its entry into the nucleus and subsequent DNA binding [54], which would down-regulate slow-fiber gene expression. In our study, *Gsk3*β was downregulated in the gSkM of HFD-ED compared with HFD-corn oil animals, indicating little Nfat phosphorylation and thus, conditions that would favor production of slow-fiber type. Resistance training, testosterone treatment, growth hormone, or dehydroepiandrosterone interventions have shown favorable effects in retaining muscle mass and function [19,55,56,57,58]. However, long-term use of growth hormone treatment can be harmful due to associated side effects, making this a poor choice for intervention against muscle loss. Our findings suggest a mechanistic basis for the human results and supports the case for further research on either ED or fish oil supplements as potential molecules for the long-term prevention of sarcopenia. In this study we were unable to determine if muscle fiber type regulation is due to the effects of increased β-oxidation, or a direct effect of EPA or DHA on gene transcription. 

Although the effects of EPA and DHA on gSkM gene expression are conclusive, due to the limited sample amount we have been unable to validate many of the possible physiological effects found from transcriptome analysis. Ideally, future work should study these effects across a wider range of SkM and perform histology to confirm the hypothesis on fiber-type regulation. Similarly, our results suggest an effect on many parameters related to energy metabolism, supported in part by finding a lower fat content in the gSkM of HFD-ED fed mice compared with HFD-corn oil. Future work will need to see if this effect extends to improved glucose disposal which could extend the findings to possible relevance for T2D. A role for regulation of calcium concentrations for signaling should also be tested using muscle cell models. Future work will also need to test if these pathways are still relatively upregulated with lower proportions of *n*-3 fatty acids and at lower amounts, to test if they are relevant at normal amounts of dietary fat. 

These results clearly demonstrate the wide-ranging impact a difference in dietary fatty acid composition can have on SkM gene-transcription. It is unclear if ED regulates all of these gene targets simultaneously, or if some are a result of downstream interactions with initial ED targets. Given the clear sequential effects on energy metabolism from β-oxidation to oxidative phosphorylation, it appears likely that the observed upregulation of these processes is directly related to increased circulating EPA and DHA. Further studies on cell and rodent models of ageing and functional analysis of SkM are required to confirm these effects and the likely regulatory nodes for these mechanisms. 

## 5. Conclusions

The skeletal muscle transcriptome from mice fed HFDs differing in fat composition for 8 weeks, showed marked differences in expression of genes coding for metabolic proteins. Our results suggest muscle-protective effects of EPA and DHA against catabolic degradation, possibly via stimulation of pathways that increase mitochondrial β-oxidation capacity and control calcium release for muscle contraction. These shifts in gene expression, even against a background of HFD are clearly favorable for hindering molecular progression towards muscle loss. With the limited availability of clinical treatments for preventing muscle loss, increased intake of fatty fish that are abundant in EPA and DHA, or supplementation with marine fatty acids could provide a safe and economic alternative to pharmaceutical interventions.

## Figures and Tables

**Table 1:** Changes in Body Weight Composition and Plasma Lipid Composition.

Parameter	Control	HFD-ED	HFD-Corn oil
Total number of animals; *n*	9	12	12
Initial body weight (g)	27.5 ± 0.8	28.6 ± 0.8	24.3 ± 0.5
Final body weight (g)	31.4 ± 1	33.8 ± 0.8	36.2 ± 0.9
Change in body weight (g)	3.9 ± 0.5 ^a^	5.2 ± 0.3 ^a^	8.6 ± 0.5 ^b^
plasma cholesterol (mmol/L)	5.6 ± 0.4	5.5 ± 0.4	5.2 ± 0.6
plasma triglyceride (mmol/L)	0.9 ± 0.1 ^a,b^	1.1 ± 0.1 ^a^	0.7 ± 0.1 ^b^

The data are shown as mean ± SEM; different letters show significant different tested by ANOVA followed by Tukey’s multiple comparison test. To calculate the changes in body weight (g), initial body weight values from individual animals was subtracted from the final body weight measurement.

**Table 2:** Diet Compositions Namely Control, HFD-ED and HFD-Corn Oil (CO) [21].

Ingredient (g/100 g Diet)		Control	HFD-ED	HFD-CO
Protein	Casein	22.2	25.6	25.6
Carbohydrates	Sucrose	5.0	10.0	10.0
	Corn starch	56.0	34.8	34.8
	Cellulose	5.0	5.8	5.8
Fat	Total	5.0	15.0	15.0
	Corn oil	2.5	3.0	5.0
	Coconut oil	2.5	10.0	10.0
	EPAX oils ^a^	0	2.0	0
Minerals ^b^		2.0	2.5	2.5
Miconutrients ^c^		3.0	3.0	3.0
Choline bitartrate		1.6	2.0	2.0
Cholesterol		0	1.0	1.0
Methionine		0.2	0.3	0.3
Energy content (kJ/100 g)		1599	1752	1752
Protein E%		24	25	25
Carbohydrate E%		65	44	44
Fat E%		12	32	32
Fatty acid composition ^d^ (mg/g diet)	C10:0	0.20	1.47	1.33
	C12:0	2.37	7.58	7.72
	C14:0	1.54	4.58	4.78
	C16:0	1.90	3.44	3.59
	C18:0	0.68	2.26	2.49
	SFA	6.70	19.33	19.91
	C18:1*n*9	2.82	4.80	5.26
	MUFA	2.82	4.80	5.26
	C18:2*n*6	3.62	5.03	7.36
	C18:3*n*6	0.12	0.22	0.26
	*n*-6 PUFA	3.74	5.26	7.62
	C20:5*n*3 (EPA)	0.00	2.03	0.01
	C22:6*n*3 (DHA)	0.00	4.58	0.01
	*n*-3 PUFA	0.00	6.61	0.02

^a^ EPAX 1050. EPAX 6015. ^b^ CaCO_3_ (57.7%); KCl (19.9%); KH_2_PO_4_ (11.9%); MgSO_4_ (10.4%). ^c^ Corn starch (98.22%); Ca(IO_3_)_2_ (0.0007%); CoCO_3_ (0.064%); CuO (0.02%); FeSO_4_ (0.5%); MnO_2_ (0.035%); Na_2_MoO_4_ (0.001%); NaSeO_3_ (0.0007%); ZnO (0.1%); Vitamin A (0.013%); B_2_ (Riboflavin-5-phosphate sodium; 0.027%); B_3_ (0.1%); B_5_ (Ca Pantothenate; 0.057%); B_6_ (0.023%); B_7_ (0.0007%); B_9_ (0.007%); B_12_ (0.00008%); D_3_ (0.007%); E (0.25%); K (0.003%). ^d^ Analyses were performed in triplicate and data was obtained by Gas chromatography mass spectroscopy.

**Table 3:** The Fatty Acid Profiles of Different Lipid Fractions in Gastrocnemicus Skeletal Muscle.

Lipid Fraction	mg/g	mg/g	mg/g	% value	% value	% value
**Neutral lipids**	**Control**	**HFD-ED**	**HFD-Corn oil**	**Control**	**HFD-ED**	**HFD-Corn oil**
C12:0	0.12 ± 0.06 ^a^	1.51 ± 0.42 ^b^	2.87 ± 0.41 ^c^	0.44 ± 0.18 ^a^	3.93 ± 0.21 ^b^	4.02 ± 0.33 ^b^
C14:0	1.18 ± 0.24 ^a^	3.15 ± 0.76	4.81 ± 0.49 ^b^	4.35 ± 0.48 ^a^	8.19 ± 0.52 ^b^	6.74 ± 0.23 ^c^
C16:0	8.11 ± 2.31 ^a^	9.75 ± 2.28 ^a^	19.01 ± 2.11 ^b^	29.93 ± 1.78	25.35 ± 1.51	26.63 ± 2.34
C18:0	0.55 ± 0.05	0.65 ± 0.05	0.68 ± 0.04	2.03 ± 0.48 ^a^	1.69 ± 0.4 ^a^	0.95 ± 0.09 ^b^
C18:1	13.11 ± 3.05 ^a^	14.86 ± 3.45 ^a^	29.2 ± 2.19 ^b^	48.38 ± 0.81 ^a^	38.64 ± 1.06 ^b^	40.91 ± 1.6 ^b^
C18:2 *n*6	3.75 ± 0.78 ^a^	7.29 ± 1.89 ^a^	14.55 ± 1.25 ^b^	13.84 ± 0.67 ^a^	18.95 ± 0.9 ^b^	20.38 ± 0.73 ^b^
C18:3 *n*3	0.07 ± 0.04	0.07 ± 0.03	0.09 ± 0.02	0.26 ± 0.08	0.18 ± 0.04	0.13 ± 0.03
C20:3 *n*6	0.02 ± 0.01	0.01 ± 0 ^a^	0.03 ± 0 ^b^	0.07 ± 0.06	0.03 ± 0	0.04 ± 0
C22:5 *n*3	0.03 ± 0.01	0.06 ± 0.01	0 ± 0	0.11 ± 0.09	0.16 ± 0.01 ^a^	0 ± 0 ^b^
C22:6 *n*3	0.14 ± 0.02 ^a^	1.1 ± 0.09 ^b^	0.14 ± 0.02 ^a^	0.52 ± 0.25 ^a^	2.86 ± 0.68 ^b^	0.2 ± 0.04 ^a^
Total neutral lipids	27.1 ± 6.48 ^a^	38.46 ± 8.89 ^a^	71.38 ± 6.13 ^b^			
**Free fatty acids**	**Control**	**HFD-ED**	**HFD-Corn oil**	**Control**	**HFD-ED**	**HFD-Corn oil**
C12:0	0.13 ± 0.04	0.16 ± 0.04 ^a^	0.04 ± 0.01 ^b^	6.6 ± 2.11	8.21 ± 2.26 ^a^	1.61 ± 0.43 ^b^
C16:0	0.52 ± 0.07 ^a^	0.64 ± 0.02	0.78 ± 0.04 ^b^	26.4 ± 3.14 ^a^	32.82 ± 1.34 ^b^	31.33 ± 2.03 ^b^
C16:1	0.12 ± 0.03	0.09 ± 0.03	0.15 ± 0.05	6.09 ± 1.17	4.62 ± 1.3	6.02 ± 1.58
C18:1	1.07 ± 0.09	0.89 ± 0.07 ^a^	1.29 ± 0.12 ^b^	54.31 ± 4.03 ^a^	45.64 ± 1.96 ^b^	51.81 ± 1.68
C18:2 *n*6	0 ± 0 ^a^	0 ± 0 ^a^	0.11 ± 0.02 ^b^	0 ± 0 ^a^	0 ± 0 ^a^	4.42 ± 0.46 ^b^
C18:3 *n*3	0.09 ± 0	0.07 ± 0.01	0.07 ± 0.01	4.57 ± 0.64 ^a^	3.59 ± 0.51	2.81 ± 0.27 ^b^
C20:3 *n*3	0.04 ± 0 ^a^	0 ± 0 ^b^	0.04 ± 0.01 ^a^	2.03 ± 0.27 ^a^	0 ± 0 ^b^	1.61 ± 0.24 ^c^
C22:6 *n*3	0 ± 0 ^a^	0.09 ± 0.03 ^b^	0 ± 0 ^a^	0 ± 0 ^a^	4.62 ± 1.25 ^b^	0 ± 0.14 ^a^
Total free fatty acids	1.97 ± 0.17	1.95 ± 0.12	2.49 ± 0.21			
**Phospholipids**	**Control**	**HFD-ED**	**HFD-Corn oil**	**Control**	**HFD-ED**	**HFD-Corn oil**
C14:0	0.33 ± 0.02 ^a^	0.83 ± 0.04 ^b^	0.64 ± 0.07	2.17 ± 0.14 ^a^	6.14 ± 0.28 ^b^	4.7 ± 0.49 ^c^
C16:0	5.06 ± 0.06 ^a^	4.93 ± 0.09	4.73 ± 0.06 ^b^	33.29 ± 0.76 ^a^	36.49 ± 0.55 ^b^	34.75 ± 1.37
C18:0	1.97 ± 0.33	2.65 ± 0.18	2.01 ± 0.26	12.96 ± 2.38	19.62 ± 1.41	14.77 ± 2.02
C18:1	3.67 ± 0.68 ^a^	0.39 ± 0.26 ^b^	2.07 ± 0.44 ^a^	24.14 ± 3.71 ^a^	2.89 ± 1.81 ^b^	15.21 ± 3.12 ^a^
C18:2 *n*6	1.73 ± 0.06 ^a^	0.58 ± 0.09 ^b^	2.13 ± 0.05 ^c^	11.38 ± 0.28 ^a^	4.29 ± 0.61 ^b^	15.65 ± 0.6 ^c^
C20:3 *n*6	0.05 ± 0 ^a^	0 ± 0 ^b^	0.06 ± 0.01 ^a^	0.33 ± 0 ^a^	0 ± 0 ^b^	0.44 ± 0.04 ^a^
C20:4 *n*6	0.57 ± 0.01 ^a^	0.1 ± 0 ^b^	0.55 ± 0.01 ^a^	3.75 ± 0.12 ^a^	0.74 ± 0.01 ^b^	4.04 ± 0.28^a^
C20:5 *n*3	0 ± 0	0.04 ± 0	0.02 ± 0.02	0 ± 0	0.3 ± 0.01	0.15 ± 0.15
C22:3 *n*6	0.54 ± 0.02 ^a^	0.07 ± 0 ^b^	0.26 ± 0.03 ^c^	3.55 ± 0.18 ^a^	0.52 ± 0.01 ^b^	1.91 ± 0.23 ^c^
C22:5 *n*3	0.05 ± 0 ^a^	0.11 ± 0 ^b^	0.06 ± 0.01 ^a^	0.33 ± 0.02 ^a^	0.81 ± 0.02 ^b^	0.44 ± 0.05 ^c^
C22:6 *n*3	1.23 ± 0.03 ^a^	3.45 ± 0.06 ^b^	1.07 ± 0.1 ^a^	8.09 ± 0.3 ^a^	25.54 ± 0.53 ^b^	7.86 ± 0.73 ^a^
Total phospholipids	15.2 ± 0.47 ^a^	13.15 ± 0.34 ^b^	13.61 ± 0.45 ^b^			
Total Fat content	44.26 ± 6.99	53.56 ± 9	87.47 ± 6.45			

The fatty acid profiles from the mice fed, either control, HFD-corn oil or HFD-ED are shown as mean ± SEM and as a proportion of total fatty acid fraction; different letters show statistical difference tested by ANOVA followed by Tukey’s multiple comparison test. For details see methods section.

## Supplementary Materials

The following are available online at www.mdpi.com/link, Figure S1: Protein lysate from the gSkM of HFD-ED and HFD-corn oil were analyzed by Western blot using antibodies for total Acc, p-Acc (Ser79) and total anti-Troponin C1. Gapdh was used as a loading control for the Western blot, Figure S2: (a) Principle Component Analysis (PCA) plot based on the normalized gene expression from the gSkM tissue fed control, HFD-ED and HFD-corn oil is plotted for assessing the quality of the datasets. No animals or any related data was excluded from further assessment. (b) Venn diagrams showing the number of differentially expressed genes (FDR adjusted *p*-value < 0.001) in each diet-comparison for gSkM tissue. HFD-ED compared with control diet and HFD-corn oil fed mice shows most transcriptional response to the diet. Key: ED = HFD-ED; CO = HFD-corn oil and CD = control diet, Figure S3: (a) Fatty acid β-oxidation pathway rendered by the pathview function from the Kegg pathway database. Genes highlighted in red are upregulated for the comparison HFD-ED vs. HFD-corn oil. (b) Krebs cycle pathway rendered by the pathview function from the Kegg pathway database. Genes highlighted in red are upregulated for the comparison HFD-ED vs. HFD-corn oil. (c) Oxidative phosphorylation pathway rendered by the pathview function from the Kegg pathway database. Genes highlighted in red are upregulated for the comparison HFD-ED vs. HFD-corn oil, Table S1: Table for the selection criteria for animals for microarray analysis. Animals marked in green were selected for microarray analysis, Table S2: The top 100 differentially regulated genes regulated by HFD-ED compared to HFD-corn oil fed mice, based on adjusted *p*-value.
