# Supplementary material for: Eicosapentaenoic and Docosahexaenoic Acid-Enriched High Fat Diet Delays Skeletal Muscle Degradation in Mice
Source: Nutrients. 2016 Sep 3;8(9):543. doi: 10.3390/nu8090543 (PMC5037530; doi:10.3390/nu8090543)
Supplement: Supplementary file 1 [file nutrients-08-00543-s001.docx]

Supplementary Materials: Eicosapentaenoic and Docosahexaenoic Acid-Enriched High Fat Diet Delays Skeletal Muscle Degradation in Mice

Nikul K. Soni, Alastair B. Ross, Nathalie Scheers, Otto I. Savolainen, Intawat Nookaew, Britt G. Gabrielsson and Ann-Sofie Sandberg

**Figure S1.** Protein lysate from the gSkM of HFD-ED and HFD-corn oil were analyzed by Western blot using antibodies for total Acc, p-Acc (Ser79) and total anti-Troponin C1. Gapdh was used as a loading control for the Western blot.

|  |
| --- |
| (a) |
| 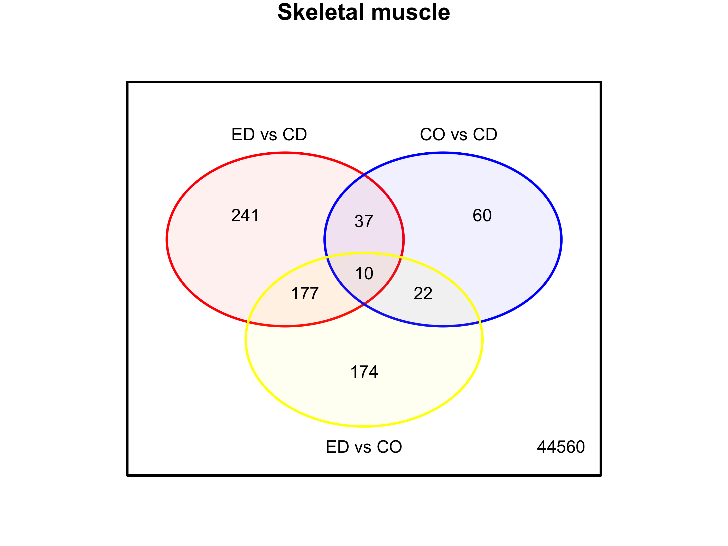 |
| (b) |

**Figure S2.** (**a**) Principle Component Analysis (PCA) plot based on the normalized gene expression from the gSkM tissue fed control, HFD-ED and HFD-corn oil is plotted for assessing the quality of the datasets. No animals or any related data was excluded from further assessment. (**b**) Venn diagrams showing the number of differentially expressed genes (FDR adjusted *p*-value < 0.001) in each diet-comparison for gSkM tissue. HFD-ED compared with control diet and HFD-corn oil fed mice shows most transcriptional response to the diet. Key: ED = HFD-ED; CO = HFD-corn oil and CD = control diet.

|  |
| --- |
| (a) |
|  |
| (b) |
|  |
| (c) |

**Figure S3.** (**a**) Fatty acid β-oxidation pathway rendered by the pathview function from the Kegg pathway database. Genes highlighted in red are upregulated for the comparison HFD-ED vs. HFD-corn oil. (**b**) Krebs cycle pathway rendered by the pathview function from the Kegg pathway database. Genes highlighted in red are upregulated for the comparison HFD-ED vs. HFD-corn oil. (**c**) Oxidative phosphorylation pathway rendered by the pathview function from the Kegg pathway database. Genes highlighted in red are upregulated for the comparison HFD-ED vs. HFD-corn oil.

**Table S1.** Table for the selection criteria for animals for microarray analysis. Animals marked in green were selected for microarray analysis.

| **Animals group** | **Animal ID** | **Body weight (g)** | **plasma TG (mM)** | **plasma chol. (mM)** |
| --- | --- | --- | --- | --- |
| Control | 3527 | 31.2 | 1.24 | 8.2 |
| Control | 7343 | 37.4 | 0.92 | 4.8 |
| Control | 5122 | 29.8 | 1.12 | 6,0 |
| Control | 7037 | 30,0 | 0.52 | 4.6 |
| Control | 3377 | 28.1 | 1,00 | 4.8 |
| Control | 0311 | 28.4 | 0.88 | 6.4 |
| Control | 4356 | 33,0 | 1.12 | 5.2 |
| Control | 5807 | 32.8 | 0.72 | 5.2 |
| Control | 0104 | 31.6 | 0.4 | 4.8 |
| HFD_ED | 2824 | 30.5 | 0.92 | 5.4 |
| HFD_ED | 0864 | 26.7 | 1.12 | 7,0 |
| HFD_ED | 6534 | 30 | 0.84 | 6,0 |
| HFD_ED | 7887 | 30.3 | 0.68 | 4.8 |
| HFD_ED | 6304 | 30.2 | 1.04 | 5.4 |
| HFD_ED | 7011 | 23.5 | 0.92 | 6.4 |
| HFD_ED | 5345 | 31.1 | 1.2 | 4.2 |
| HFD_ED | 6572 | 29.4 | 2,00 | 4.6 |
| HFD_ED | 2790 | 27.1 | 1.16 | 6,0 |
| HFD_ED | 5304 | 32 | 1.16 | 5.2 |
| HFD_ED | 7538 | 27.4 | 1.16 | 8,0 |
| HFD_ED | 4869 | 24.7 | 1,00 | 2.8 |
| HFD_corn oil | 1787 | 26 | 0.64 | 4 |
| HFD_corn oil | 9895 | 25.2 | 0.48 | 3.2 |
| HFD_corn oil | 6524 | 21.3 | 0.56 | 3.6 |
| HFD_corn oil | 5592 | 24.3 | 0.44 | 3 |
| HFD_corn oil | 5008 | 24.8 | 0.56 | 5 |
| HFD_corn oil | 3014 | 24.4 | 0.84 | 6.4 |
| HFD_corn oil | 6523 | 25.9 | 0.72 | 5.6 |
| HFD_corn oil | 9101 | 25 | 0.64 | 5.4 |
| HFD_corn oil | 3358 | 27 | 0.92 | 6 |
| HFD_corn oil | 2539 | 23.5 | 0.72 | 7 |
| HFD_corn oil | 1091 | 22.2 | 1.12 | 10 |
| HFD_corn oil | 1088 | 22.2 | 0.4 | 3.6 |

**Table S2.** The top 100 differentially regulated genes regulated by HFD-ED compared to HFD-corn oil fed mice, based on adjusted *p*-value.

| **Symbol** | **Probe_Id** | **logFC** | **AveExpr** | **adj.P.Val** |
| --- | --- | --- | --- | --- |
| scl0002124.1_39 | ILMN_2519944 | 1.03 | 8.03 | 3.00E-18 |
| Mb | ILMN_1234662 | 0.68 | 8.77 | 4.29E-18 |
| Tnnc1 | ILMN_2503052 | 1.05 | 8.31 | 3.43E-18 |
| Myl2 | ILMN_1260428 | 1.21 | 8.72 | 3.43E-18 |
| 9830108D13Rik | ILMN_1241083 | 1.00 | 7.43 | 3.00E-18 |
| Sln | ILMN_2918875 | 0.70 | 7.43 | 4.97E-17 |
| Myoz2 | ILMN_1234857 | 0.90 | 8.01 | 4.14E-16 |
| Myl3 | ILMN_2691780 | 0.90 | 8.73 | 5.00E-15 |
| Mbp | ILMN_2737200 | 0.78 | 8.08 | 5.00E-15 |
| Ampd1 | ILMN_2971142 | -0.41 | 8.63 | 3.23E-14 |
| Scd1 | ILMN_1237375 | -1.33 | 13.33 | 7.41E-14 |
| scl0002069.1_48 | ILMN_2443164 | 0.72 | 8.31 | 6.67E-14 |
| Fndc5 | ILMN_3107467 | 0.47 | 7.45 | 7.41E-14 |
| Pitx2 | ILMN_3118071 | 0.66 | 7.62 | 7.41E-14 |
| Cntnap2 | ILMN_2681321 | -0.61 | 7.25 | 9.57E-14 |
| A730024G14Rik | ILMN_2575694 | -0.37 | 7.68 | 1.78E-13 |
| Plcd4 | ILMN_3062126 | -0.36 | 7.35 | 9.57E-14 |
| LOC226017 | ILMN_1245043 | 0.99 | 8.75 | 2.98E-13 |
| 8030451F13Rik | ILMN_2744469 | 0.73 | 8.24 | 6.44E-13 |
| Ihpk3 | ILMN_2600779 | 0.37 | 7.37 | 6.03E-13 |
| Lmcd1 | ILMN_2628940 | 0.58 | 7.46 | 4.92E-13 |
| Htra4 | ILMN_2669289 | -0.76 | 7.39 | 1.94E-13 |
| B230312C02Rik | ILMN_1236176 | -0.26 | 7.15 | 3.82E-12 |
| Tpm2 | ILMN_2787172 | 0.42 | 7.62 | 4.89E-12 |
| Prkab2 | ILMN_1215061 | -0.45 | 7.78 | 6.62E-12 |
| Myh1 | ILMN_2740259 | 0.38 | 7.34 | 9.92E-12 |
| Itgb6 | ILMN_2805375 | 0.26 | 7.61 | 1.36E-11 |
| EG433229 | ILMN_2660466 | 0.93 | 9.97 | 6.62E-12 |
| Tceal7 | ILMN_2715279 | -0.34 | 7.55 | 1.50E-11 |
| Smtnl1 | ILMN_2774281 | 0.60 | 7.50 | 2.16E-11 |
| Prkag3 | ILMN_2468772 | -0.47 | 7.70 | 1.72E-11 |
| Taf7 | ILMN_2748412 | -0.19 | 7.11 | 3.80E-11 |
| Myh2 | ILMN_1219423 | 0.51 | 8.91 | 3.80E-11 |
| Myh7 | ILMN_2945491 | 0.32 | 7.22 | 7.10E-11 |
| Prkag3 | ILMN_3092923 | -0.34 | 7.23 | 8.09E-11 |
| A530082L16Rik | ILMN_1250557 | 0.34 | 7.23 | 1.25E-10 |
| Barx2 | ILMN_2437728 | 0.46 | 7.28 | 8.09E-11 |
| Esrrb | ILMN_1228497 | 0.52 | 7.39 | 1.96E-10 |
| Csrp3 | ILMN_2789651 | 0.71 | 7.80 | 2.76E-10 |
| H19 | ILMN_2906728 | 0.57 | 7.83 | 2.38E-10 |
| Ociad2 | ILMN_2943722 | -0.42 | 8.01 | 4.50E-10 |
| Actc1 | ILMN_2598916 | 0.56 | 7.93 | 2.98E-10 |
| Hoxd9 | ILMN_2800183 | -0.31 | 7.50 | 5.51E-10 |
| Rasd2 | ILMN_1223875 | -0.46 | 8.03 | 2.88E-10 |
| Myh1 | ILMN_2865744 | 0.54 | 8.38 | 7.64E-10 |
| Actc1 | ILMN_2767216 | 0.46 | 7.40 | 4.62E-10 |
| Pygm | ILMN_1220498 | -0.34 | 8.28 | 1.04E-09 |
| 8030451F13Rik | ILMN_2969105 | 0.54 | 8.15 | 8.46E-10 |
| Tnnt1 | ILMN_2460136 | 0.95 | 8.53 | 7.79E-10 |
| Arpp21 | ILMN_1241866 | 0.41 | 7.25 | 1.04E-09 |
| 2310040G07Rik | ILMN_1232245 | -0.28 | 7.49 | 1.89E-09 |
| LOC100048376 | ILMN_1241150 | 0.42 | 7.46 | 2.14E-09 |
| 2610304F08Rik | ILMN_1246824 | -0.20 | 7.35 | 1.96E-09 |
| Actn2 | ILMN_2764727 | 0.54 | 9.11 | 2.44E-09 |
| Grb14 | ILMN_2625280 | 0.52 | 7.88 | 3.24E-09 |
| Maf | ILMN_1237448 | -0.20 | 7.27 | 3.36E-09 |
| Fndc5 | ILMN_2763060 | 0.40 | 7.56 | 3.04E-09 |
| Clip4 | ILMN_2606470 | 0.46 | 7.98 | 4.98E-09 |
| Kcnab1 | ILMN_2915671 | -0.35 | 7.37 | 5.22E-09 |
| Myoz1 | ILMN_2615250 | -0.32 | 8.60 | 5.97E-09 |
| Mylc2pl | ILMN_2732795 | 0.25 | 7.13 | 6.22E-09 |
| Prei4 | ILMN_1251682 | -0.36 | 7.56 | 6.70E-09 |
| Plekha4 | ILMN_1243826 | 0.41 | 7.73 | 7.09E-09 |
| Svil | ILMN_2675800 | -0.22 | 7.21 | 6.94E-09 |
| Epm2a | ILMN_2920454 | -0.19 | 7.14 | 8.44E-09 |
| Ttn | ILMN_1231542 | -0.33 | 7.87 | 8.89E-09 |
| Chrna1 | ILMN_1245221 | 0.26 | 7.45 | 1.15E-08 |
| Atp2a2 | ILMN_1239742 | 0.89 | 10.09 | 1.21E-08 |
| Fhl2 | ILMN_2770386 | 0.22 | 7.20 | 1.22E-08 |
| LOC381283 | ILMN_1233840 | 0.34 | 7.31 | 1.24E-08 |
| H19 | ILMN_1256343 | 0.59 | 8.32 | 9.96E-09 |
| Ankrd2 | ILMN_2806065 | 0.87 | 7.98 | 9.69E-09 |
| Bdh1 | ILMN_1231553 | 0.82 | 8.10 | 1.29E-08 |
| Lep | ILMN_2695964 | -1.61 | 9.26 | 1.42E-08 |
| Tnni1 | ILMN_2977056 | 0.39 | 7.37 | 1.36E-08 |
| 2310002L09Rik | ILMN_1214119 | 0.30 | 8.04 | 1.39E-08 |
| Camk2a | ILMN_2677393 | -0.34 | 7.38 | 1.61E-08 |
| scl0001978.1_6 | ILMN_2435584 | 0.67 | 8.47 | 1.63E-08 |
| Adipoq | ILMN_2738082 | -1.04 | 10.48 | 1.63E-08 |
| Pdlim3 | ILMN_1235230 | -0.35 | 8.35 | 1.80E-08 |
| Actn2 | ILMN_2797061 | 0.59 | 8.63 | 9.46E-09 |
| 8030451F13Rik | ILMN_2739266 | 0.26 | 7.20 | 2.32E-08 |
| Myom3 | ILMN_2673358 | 1.05 | 7.95 | 3.22E-08 |
| Kcnab1 | ILMN_1216469 | -0.26 | 7.26 | 3.45E-08 |
| Aph1a | ILMN_2588759 | 0.29 | 7.79 | 3.63E-08 |
| Mpz | ILMN_2817329 | 0.35 | 7.34 | 3.58E-08 |
| Prx | ILMN_2593225 | 0.23 | 7.16 | 3.94E-08 |
| AW120700 | ILMN_1260036 | -0.78 | 7.72 | 2.43E-08 |
| 1110049N09Rik | ILMN_2543459 | -0.40 | 7.51 | 2.39E-08 |
| 8430427K15 | ILMN_1256147 | 0.17 | 7.14 | 4.85E-08 |
| LOC100041585 | ILMN_1249590 | -0.25 | 7.35 | 6.21E-08 |
| Myh6 | ILMN_2788836 | 0.30 | 7.25 | 6.14E-08 |
| Rbbp7 | ILMN_2896823 | -0.21 | 7.22 | 6.58E-08 |
| Vgll2 | ILMN_2495753 | 0.51 | 7.75 | 5.71E-08 |
| Kcnma1 | ILMN_1244166 | 0.24 | 7.25 | 7.35E-08 |
| Art5 | ILMN_2890301 | 0.40 | 7.63 | 7.38E-08 |
| Aoc3 | ILMN_2625920 | -0.68 | 10.18 | 8.77E-08 |
| Dtna | ILMN_1221805 | 0.31 | 7.74 | 8.77E-08 |
| Mylk2 | ILMN_1257739 | -0.27 | 7.45 | 8.94E-08 |
| Mib1 | ILMN_2933834 | -0.37 | 7.55 | 9.59E-08 |
